# Total Joint Arthroplasty in Patients With Lymphedema as Compared to a Propensity-Matched Control Cohort

**DOI:** 10.1016/j.artd.2023.101307

**Published:** 2023-12-30

**Authors:** William H. Cusma, Nicholas M. Brown, William J. Hopkinson

**Affiliations:** a Department of Orthopaedics and Rehabilitation, Stritch School of Medicine, Maywood, IL, USA; b Department of Orthopaedics and Rehabilitation, Loyola University Medical Center, Maywood, IL, USA; c Department of Orthopaedics and Rehabilitation, Maywood, IL, USA

**Keywords:** Arthroplasty, Knee, Hip, Lymphedema, Complications, Infection

## Abstract

**Background:**

Lymphedema is rare in arthroplasty patients but has been associated with a higher complication rate. This study sought to determine the outcomes of total joint arthroplasty in patients with lymphedema as compared to a matched control cohort.

**Methods:**

Treatment-control propensity matching was implemented on 335 patients following total knee or hip arthroplasty generating 5-patient sets of one patient with presurgery lymphedema (67 total) to 4 patients without presurgery lymphedema (268 total) and matched on age, sex, and surgery year. Body mass index and presence of diabetes were controlled using multivariable generalized estimating equations.

**Results:**

In the lymphedema cohort, 1 patient (1.5%) had a deep vein thrombosis within 90 days of their surgery, 36 (53.7%) were discharged to a rehabilitation center, 16 (23.9%) had a readmission, 14 (20.9%) were admitted to the emergency room within 90 days, 6 (9.0%) experienced infection, and 6 (9.0%) had a revision/reoperation. Lymphedema significantly increased emergency room admission within 90 days (odds ratio [OR] 4.56, *P* = .01) and non-home discharge (OR 4.14, *P* < .01), affected readmission within 90 days (OR 2.21, *P* = .09), revision/reoperation (OR 2.82, *P* = .09), and no effect on deep vein thrombosis within 90 days (OR 0.57, *P* = .45), postsurgical infection (OR 1.47, *P* = .45), length of stay (OR 0.00, *P* = .99), operative time (OR 0.04, *P* = .38), or estimated blood loss (OR 0.09, *P* = .47), after adjusting for various factors.

**Conclusions:**

Preoperative lymphedema is a significant risk factor for patients who are undergoing total joint arthroplasty. Preoperative and postoperative modalities should be utilized to help control lymphedema and mitigate these increased risks.

## Introduction

Lymphedema is an uncommon comorbidity in arthroplasty patients but has been associated with a higher complication rate [1]. Common causes of arthroplasty complications experienced by patients with lymphedema include periprosthetic infection, deep venous thrombosis, and subsequent revision [2]. Lymphedema is characterized by damage or blockage to the lymphatic vessels, resulting in progressive and chronic high interstitial edema. Risk factors for this condition include age, obesity, radiation therapy, and previous surgery.

Kolz et al demonstrated that lymphedema significantly increased the risk of complications following knee replacement with a hazard ratio of 2.87 comparing reoperation in patients who were determined to be healthy prior to their surgery to those who had been diagnosed with lymphedema presurgery [3]. Additionally, this study demonstrated a postoperative infection hazard ratio of 2.37 when comparing the same 2 groups. Rainer et al investigated primary total hip arthroplasty using similar methodology and reported an increased risk of reoperation (hazard ratio 3.16) and postoperative infection (hazard ratio 4.48) [1]. While the majority of research has shown an increased risk of complications in this population, there are a limited number of studies specific to primary joint replacement with relatively low patient numbers. Therefore, the exact complication profile and risk level have not been fully determined.

The purpose of this study was to compare the complication rate, length of stay, and discharge location in patients with lower extremity lymphedema receiving total joint arthroplasty as compared to a propensity-matched cohort. Specifically, this study was designed to answer the following questions: Are patients with a preexisting lymphedema diagnosis at an increased risk of complications associated with lower extremity joint replacement such as deep vein thrombosis (DVT) and prosthetic joint infection? Does a preexisting lymphedema diagnosis contribute to a higher risk of adverse outcomes following the immediate postoperative period such as emergency room (ER) admission within 90 days of surgery, readmission, and reoperation within 90 days of surgery?

## Material and methods

This was an institutional review board-approved retrospective study that evaluated primary total hip and knee arthroplasty procedures performed at one tertiary academic center between 1/1/2005 and 10/31/2020. Using an institutional database, patients met inclusion criteria based on Current Procedural Terminology (CPT) code 27447, arthroplasty, knee and plateau, medial and lateral compartments, unilateral and 27,130 arthroplasty, acetabular and proximal, and femoral prosthetic replacement. Additionally, patients in the lymphedema cohort were identified using International Classification of Diseases - 10 (ICD-10) code I89.0, lymphedema. All patients were verified during chart review to have had lymphedema preoperatively. Patients within the lymphedema cohort who did not have a lymphedema diagnosis prior to surgery were excluded from this study. Demographic data was collected, and outcomes included length of hospital stay, estimated blood loss, discharge location, revision within 90 days of surgery, reoperation within 90 days of surgery, emergency department visit within 90 days of surgery, DVT or pulmonary embolism within 90 days of surgery, and postsurgical infection.

Frequencies and percentages are reported for categorical variables. Means and standard deviations are reported for continuous variables. Descriptive summary statistics are reported overall and stratified by lymphedema status. Standardized differences between lymphedema groups are reported [4].

Treatment-control propensity score matching was implemented on a sample of patients undergoing total knee arthroplasty or total hip arthroplasty to generate unique, 5-patient sets consisting of one patient with presurgery lymphedema to 4 patients without presurgery lymphedema, matched on age, sex, and year of surgery [5]. Multivariable generalized estimating equations estimated the adjusted effects of lymphedema on several postsurgical outcomes separately. Generalized estimating equation working correlation structures accounted for the correlation in lymphedema/nonlymphedema-matched sets. These models featured a binomial distribution and a logit link for dichotomous outcomes, a normal distribution and an identity link for continuous outcomes, and an unstructured working correlation structure in all cases. Natural log transformations were applied to all continuous outcomes to normalize their distributions. Population marginal means or odds ratio (OR) estimates, corresponding 95% confidence intervals (CIs), and type III likelihood ratio test *P*-values are reported for the effect of lymphedema on each postsurgical outcome.

This analytic sample consisted of 335 unique patients matched into 67 unique sets on age, sex, and year of surgery. Included in the lymphedema cohort were a total of 67 patients, 47 women and 20 men with an average age of 68. In the control cohort, there were 268 total patients, 190 women and 78 men with an average age of 67. (Table 1) The lymphedema cohort included 21 hip and 46 knee arthroplasties, while the nonlymphedema cohort included 99 hips and 169 knees (Table 2).

**Table 1:** Standardized differences of matched baseline covariates.

Covariate	Lymphedema (n = 67)	No lymphedema (n = 268)	Standardized difference
Age (y), Mean (SD)	67.90 (10)	66.59 (10)	0.126
Sex, n (%)			
Female	47 (70)	190 (71)	−0.016
Male	20 (30)	78 (29)	0.016

SD, standard deviation.

**Table 2:** Patient demographics by lymphedema status.

Variable	Lymphedema (n = 67)	No lymphedema (n = 268)	*P*
Race, n (%)			
Other	0	23 (9)	*P* = .006
Black	10 (15)	37 (14)	*P* = .4
White	57 (85)	208 (78)	*P* = .09
Ethnicity, n (%)			
Hispanic	0	30 (11)	*P* = .5
Non-Hispanic	67 (100)	238 (89)	
Hip arthroplasty, n (%)			
Yes	21 (31)	99 (37)	*P* = .2
No	46 (69)	169 (63)	
Knee arthroplasty, n (%)			
Yes	46 (69)	169 (63)	*P* = .2
No	21 (31)	99 (37)	

## Results

Six of 67 (9%) patients with lymphedema vs 9 of 268 (3%) control patients had a revision/reoperation. The data reflected a trend of 2.82 (1.05 to 7.56, *P* = .09) times increased odds of revision or reoperation after controlling for age, sex, diabetes status and body mass index (BMI). Six (9%) patients with lymphedema vs 16 (6%) of control patients had a postoperative infection. Similarly, in the lymphedema cohort, 1 patient (1.5%) had a DVT within 90 days of their surgery, and 9 (3%) experienced a DVT in the control cohort. Controlling for age, sex, diabetes status, and BMI, there was no effect of lymphedema status on DVT within 90 days (OR of 0.57 [CI 0.10 to 3.23, *P* = .45]), postsurgical infection (1.47 [CI 0.62 to 3.46], *P* = .45), operative time is 1.09 (CI −1.096 to 1.35) *P* = .38, or average estimated blood loss (1.23 [CI 1.32 to 1.99, *P* = .47]) (Table 3).

**Table 3:** Patient postoperative outcomes by lymphedema status.

Variable	Lymphedema (n = 67)	No lymphedema (n = 268)	Adjusted OR (95% CI)	*P*-value
ER admission within 90 d, n (%)				
Yes	14 (21)	18 (7)	4.56 (1.89, 11.00)	**.01**
No	53 (79)	250 (93)		
Readmission within 90 d, n (%)				
Yes	16 (24)	32 (12)	2.21 (1.04, 4.73)	.09
No	51 (76)	236 (88)		
Revision/reoperation, n (%)				
Yes	6 (9)	9 (3)	2.82 (1.05, 7.56)	.09
No	61 (91)	259 (97)		
DVT within 90 d, n (%)				
Yes	1 (1)	9 (3)	0.57 (0.10, 3.23)	.45
No	66 (99)	259 (97)		
Postsurgical infection, n (%)				
Yes	6 (9)	16 (6)	1.47 (0.62, 3.46)	.45
No	61 (91)	252 (94)		
Discharge destination, n (%)				
Rehabilitation	36 (54)	70 (26)	4.14 (2.21, 7.75)	**<.01**
Home	31 (46)	198 (74)		
BMI, mean (SD)	38.93 (9)	32.52 (7)		
Length of stay (d), median (IQR)	3.00 (3-3)	3.00 (2-3)	(1.78 to 1.78)	*P* = .99
Operative time (min), median (IQR)	115.00 (96-130)	106.00 (88-130)	(−1.096 to 1.35)	*P* = .38
Estimated blood loss (mL), median (IQR)	200.00 (125-400)	200.00 (100-300)	(1.32 to 1.99)	*P* = .47

IQR, interquartile range; SD, standard deviation; ER, emergency room.

Bold values indicate statistical significance (*P* < .05).

There were 16 (24%) patients in the lymphedema cohort who had a readmission following their surgery, as compared to 32 (12%) in the control cohort. Similarly, 14 (21%) of patients with lymphedema were admitted to the ER within 90 days, as compared to 18 (7%) in the control cohort. After controlling for age, sex, diabetes status, and BMI, patients with lymphedema had increased overall readmission (OR 2.21, [CI 1.04 to 4.73], *P* = .09) and increased ER admission within 90 days [OR 4.56 (CI 1.89 to 11.00, *P* = .01)] (Table 4).

**Table 4:** Adjusted effects of presurgery lymphedema on TJA clinical outcomes.

Outcome	Adjusted OR (95% CI)	*P*
DVT within 90 d	0.57 (0.10, 3.23)	.45
Postsurgical infection	1.47 (0.62, 3.46)	.45
ER admission within 90 d	4.56 (1.89, 11.00)	.01^a^
Readmission within 90 d	2.21 (1.04, 4.73)	.09
Revision/reoperation	2.82 (1.05, 7.56)	.09
Discharge destination	4.14 (2.21, 7.75)^b^	<.01^a^

Outcome	Adjusted mean difference (95% CI)^c^	*P*
Length of stay (d)	0.00 (−1.78, 1.78)	.99
Operative time (min)	1.10 (−1.10, 1.35)	.38
Estimated blood loss (mL)	1.23 (−1.31, 1.99)	.47

a Significant at α = 0.05 level.

b Logistic generalized estimating equations model rehabilitation vs home (REF) for discharge destination, and presence vs absence (REF) of the indicated outcome for all other dichotomous outcomes.

c Adjusted mean differences are calculated as (lymphedema – no lymphedema).

Thirty-six (54%) lymphedema patients were discharged to a rehabilitation center, while 70 (26%) were discharged to a rehabilitation center in the control cohort. The OR of discharge to a rehabilitation center vs home was 4.14 (CI 2.21 to 7.75, *P* < .01). However, presurgery lymphedema had no effect on patient length of stay in days with an OR of 0 (CI −1.78 to 1.78, *P* = .99).

## Discussion

The purpose of this study was to compare outcomes of total joint arthroplasty of the lower extremity in patients with presurgery lymphedema to those of a propensity-matched control cohort. Regression models were utilized to control for the potential confounding effects of BMI, diabetes status, age, sex, and year of surgery. Patients with existing lymphedema who wish to undergo total joint replacement are at an increased risk of complications that may necessitate further intervention. Presurgery lymphedema is associated with an increased rate of ER admission within 90 days of surgery and non-home postoperative discharge location. Contrary to the existing data referenced above, which showed significantly higher rates of complications such as postoperative infection and DVT, the data collected in this study suggests no significant increase in the incidence of these events within the lymphedema cohort. This study reflects that a presurgery diagnosis of lymphedema is also associated with a higher risk of readmission or revision following joint replacement. These results suggest that lymphedema remains a significant risk factor for candidates of total joint arthroplasty and should be considered in clinical decision-making.

The data demonstrates that patients with lymphedema visit the ER within 90 days of surgery at a significantly higher rate than patients within the control group. While ED admissions were not specifically evaluated in similar studies, other studies did show significantly higher rates of revision and reoperation due to various causes that necessitated admission upon their return to the hospital [[2], [1], [3]]. This may simply be due to an overall higher risk of complications in this patient cohort. However, this effect may be a result of patient awareness of their condition and increases in patient education to identify the signs and symptoms of common postoperative complications such as DVT and infection. Therefore, these patients may be more vigilant and seek care sooner during their postoperative recovery.

It is well understood that existing lymphedema creates a greater risk for delayed wound healing and subsequent infection in the extremities [6]. Rainer et al found that infection was the most common complication among patients with lymphedema who undergo joint arthroplasty, with a hazard ratio of 4.48 (*P*= <0.01) while controlling for BMI [1]. This value is considerably higher than previous studies for joint replacement among patients without lymphedema [7]. This study found no relationship between lymphedema and infection in the multivariate regression model. However, each of the 6 reoperations within 90 days of surgery was due to postsurgical infection, and the absolute incidence was 9% in this cohort. The lack of statistical difference is likely due to the high (6%) rate of infection in the relatively unhealthy control cohort and limited numbers of patients in this study. While this study did not establish a statistically significant relationship between infection and lymphedema as an isolated variable, these patients are clearly at higher risk. This highlights the importance of patient optimization, intraoperative technique, and postoperative surveillance.

The lymphedema cohort was found to have a similar DVT rate as compared to the control cohort population. This finding is lower than expected when compared to other literature on this topic. While there is a trend of higher DVT risk in patients with lymphedema in existing literature, this difference is not recorded to be significant [1]. It is unclear in other studies whether lymphedema alone is an independent risk factor for higher incidence of DVT. Obesity, measured by a BMI >30, is understood to be a causal risk factor for deep venous thrombosis [8]. To help specifically evaluate the relationship between lymphedema and DVT, BMI was included in the individual matching criteria between control and lymphedema cohorts and may have been the reason why there was no difference between the groups.

Patients with existing lymphedema were discharged to a skilled rehabilitation center at a significantly higher rate than the control group. This result was not investigated in other studies related to lymphedema and, of course, was multifactorial, but it is important to discuss with the patients for preoperative planning.

Limitations to this study are acknowledged, including its retrospective design and the relatively low frequency of patients with lymphedema who undergo total joint arthroplasty as compared to healthy controls. Secondly, this data is limited to the patients of a single tertiary care center. As a result of the limited available data pool, there was an inability to further stratify the cohorts to investigate the influence of additional patient characteristics and comorbidities, and these should be explored to predict outcomes more accurately in this population. Also, many lymphedema patients tend to have a higher BMI and comorbidities. By controlling for these variables in a regression model, this does help isolate the effect of lymphedema, but in real clinical practice, many of these patients do have above-average comorbidities and BMI. Hence, controlling for these factors in a sense decreases the true risk profile of these patients in the statistical models. Finally, this study pooled hip and knee procedures for both results and matching. The authors acknowledge the difference in complication profiles and outcomes of the procedures, but this was necessary to obtain a reasonable sample size.

## Conclusions

Preoperative lymphedema is a significant risk factor for patients who are undergoing total joint arthroplasty. While there was no difference in the incidence of infection or DVT as compared to the control cohort, the lymphedema group experienced these complications at a high rate. The lymphedema group did have statistically significant higher rates of revision, readmission, discharge to advanced care, and emergency department visits after surgery. These results point out the importance of patient optimization and allow for better counseling of patients with lymphedema. The effect of specific preoperative optimization efforts on operative success is unknown in this population and should be the focus of future studies.

## CRediT authorship contribution statement

**William H. Cusma:** Data curation, Funding acquisition, Writing – original draft, Writing – review & editing. **Nicholas M. Brown:** Conceptualization, Funding acquisition, Investigation, Methodology, Supervision, Writing – review & editing. **William J. Hopkinson:** Conceptualization, Funding acquisition, Methodology, Project administration, Resources, Writing – review & editing.

## Appendix A Supplementary data

Conflict of Interest Statement for Brown

Conflict of Interest Statement for Cusma

Conflict of Interest Statement for Hopkinson
