# Supplementary material for: Total Joint Arthroplasty in Patients With Lymphedema as Compared to a Propensity-Matched Control Cohort
Source: Arthroplast Today. 2023 Dec 30;25:101307. doi: 10.1016/j.artd.2023.101307 (PMC10801212; doi:10.1016/j.artd.2023.101307)
Supplement: Conflict of Interest Statement for Cusma [file mmc2.docx]

# CONFLICT OF INTEREST STATEMENT

***THE JOURNAL of ARTHROPLASTY***

(Adopted from the American Academy of Orthopaedic Surgeons disclosure statement)

The following form **must be filled out completely and submitted by each author (example, 6 authors, 6 forms).**

**All items require a response. If there is no relevant disclosure for a given item, enter "*None*.”**

Manuscript Title

1. Royalties from a company or supplier (The following conflicts were disclosed)

None

2. Speakers bureau/paid presentations for a company or supplier (The following conflicts were disclosed)

None

3A. Paid employee for a company or supplier (The following conflicts were disclosed)

None

3B. Paid consultant for a company or supplier (The following conflicts were disclosed)

None

3C. Unpaid consultants for a company or supplier (The following conflicts were disclosed)

None

4. Stock or stock options in a company or supplier (The following conflicts were disclosed)

None

5. Research support from a company or supplier as a Principal Investigator (The following conflicts were disclosed)

None

6. Other financial or material support from a company or supplier (The following conflicts were disclosed)

Research grant to support research awarded to author William H. Cusma by National Institutes of Health, National Heart Lung and Blood Institute (T35 HL 120835)

7. Royalties, financial or material support from publishers (The following conflicts were disclosed)

None

8. Medical/Orthopaedic publications editorial/governing board (The following conflicts were disclosed)

None

9. Board member/committee appointments for a society (The following conflicts were disclosed)

None

**Each author must sign AND print or type his/her name, date and submit a separate form**

In addition, one BLINDED Conflict of Interest form (no author names used) should be submitted per manuscript with all author disclosures.

William H. Cusma 6/23/23


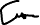

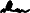

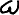


Author Name (Print or Type) Author Signature Date
